# CYP2E1 plays a suppressive role in hepatocellular carcinoma by regulating Wnt/Dvl2/β-catenin signaling

**DOI:** 10.1186/s12967-022-03396-6

**Published:** 2022-05-04

**Authors:** Lili Zhu, Xiaobei Yang, Jingyu Feng, Jian Mao, Qidong Zhang, Mengru He, Yang Mi, Yingwu Mei, Ge Jin, Haifeng Zhang

**Affiliations:** 1 Department of Biochemistry & Molecular Biology, School of Basic Medical Sciences, Zhengzhou University, 100 Kexue Road, Zhengzhou, 450001 Henan China; 2 Zhengzhou Tobacco Research Institute of China National Tobacco Company, Zhengzhou, 450001 China

**Keywords:** CYP2E1, Wnt, β-catenin, Ubiquitination, Dvl2, ROS, Hepatocellular carcinoma

## Abstract

**Objective:**

Knowledge of the role of CYP2E1 in hepatocarcinogenesis is largely based on epidemiological and animal studies, with a primary focus on the role of CYP2E1 in metabolic activation of procarcinogens. Few studies have directly assessed the effects of CYP2E1 on HCC malignant phenotypes.

**Methods:**

The expression of CYP2E1 in HCC tissues was determined by qRT-PCR, western blotting and immunohistochemistry. Overexpression of CYP2E1 in HCC cell was achieved by lentivirus transfection. The function of CYP2E1 were detected by CCK-8, wound healing, transwell assays, xenograft models and pulmonary metastasis model. TOP/FOPFlash reporter assay, western blotting, functional rescue experiments, Co-immunoprecipitation and reactive oxygen species detection were conducted to reveal the underlying mechanism of the tumor suppressive role of CYP2E1.

**Results:**

CYP2E1 expression is down-regulated in HCC tissues, and this downregulation was associated with large tumor diameter, vascular invasion, poor differentiation, and shortened patient survival time. Ectopic expression of CYP2E1 inhibits the proliferation, invasion and migration and epithelial-to-mesenchymal transition of HCC cells in vitro, and inhibits tumor formation and lung metastasis in nude mice. Mechanistic investigations show that CYP2E1 overexpression significantly inhibited Wnt/β-catenin signaling activity and decreased Dvl2 expression in HCC cells. An increase in Dvl2 expression restored the malignant phenotype of HCC cells. Notably, CYP2E1 promoted the ubiquitin-mediated degradation of Dvl2 by strengthening the interaction between Dvl2 and the E3 ubiquitin ligase KLHL12 in CYP2E1-stable HCC cells. CYP2E1-induced ROS accumulation was a critical upstream event in the Wnt/β-Catenin pathway in CYP2E1-overexpressing HCC cells.

**Conclusions:**

These results provide novel insight into the role of CYP2E1 in HCC and the tumor suppressor role of CYP2E1 can be attributed to its ability to manipulate Wnt/Dvl2/β-catenin pathway via inducing ROS accumulation, which provides a potential target for the prevention and treatment of HCC.

**Graphical Abstract:**

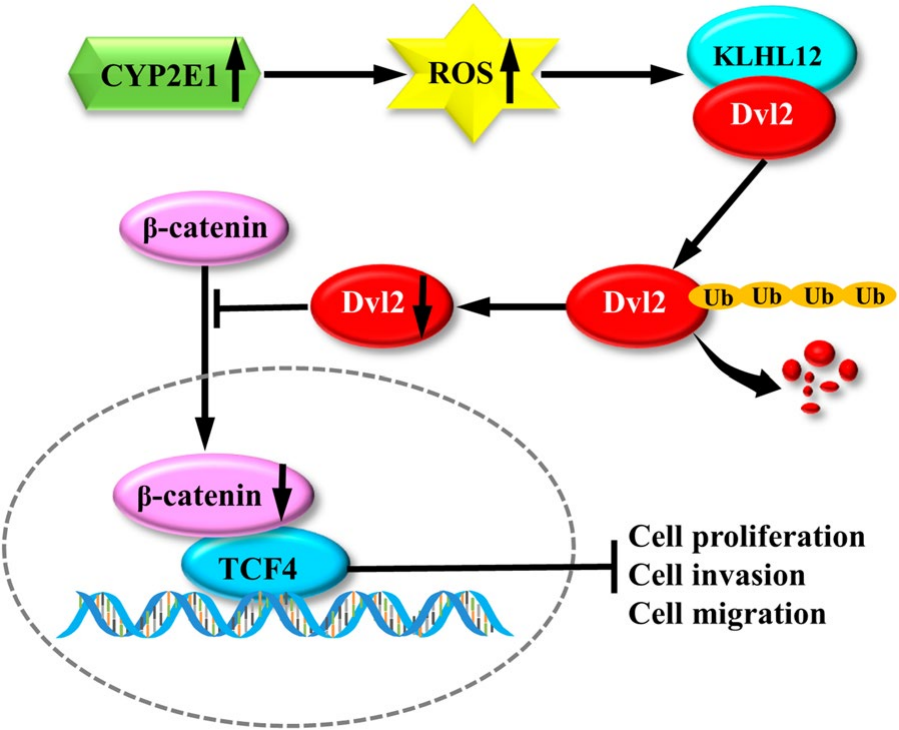

**Supplementary Information:**

The online version contains supplementary material available at 10.1186/s12967-022-03396-6.

## Introduction

Hepatocellular carcinoma (HCC) is the second leading cause of cancer-related death globally and its incidence continues to rise [1]. Currently, treatment options for advanced HCC patients are quite limited, resulting in an extremely low 5-year survival rate. HCC is associated with hepatitis B virus, hepatitis C virus, alcohol, aflatoxin and nitrosamines. However, the underlying molecular mechanisms involved in tumor formation and pathogenesis are unclear. Therefore, it is imperative to identify new molecular targets for the diagnosis and therapy of HCC.

Cytochrome P450 (CYP) enzymes are a superfamily of hemoproteins and extensively catalyze the metabolism of exogenous and endogenous compounds. CYP also exerts a crucial role in the development and progression of cancer, diabetes, heart disease, and Parkinson's disease [2–5]. CYP2E1 is the most abundant CYP isoform in human liver and is the main enzyme that metabolizes a number of low molecular weight compounds, such as ethanol, acetaminophen, benzene, and carbon tetrachloride, and cancer suspects like nitrosamines and azo compounds [6, 7]. Owing to its contribution to the metabolic activation of procarcinogens, many studies have explored the relevance of CYP2E1 to HCC susceptibility. For example, CYP2E1 catalyzes alcohol oxidation to acetaldehyde under long-term consumption of ethanol, and acetaldehyde forms DNA adducts, which may play an important role in hepatocarcinogenesis in patients with alcoholic liver injury [8]. In addition, CYP2E1 converts diethylnitrosamine to genotoxic products which damage DNA irreversibly, leading to hepatocarcinogenesis in rat studies.

However, the most common etiology of HCC is viral infection (hepatitis B virus and hepatitis C virus), not procarcinogens. Especially in China, 85% of HCC cases are associated with chronic HBV infection [9, 10]. Our knowledge of CYP2E1 in non-procarcinogen-related HCC is limited. Recently, several groups independently revealed that both the expression level and the activity of CYP2E1 are decreased in HCC as compared to adjacent normal liver tissues [11, 12]. From liver lesion and cirrhosis to the tumor occurrence, in the rat HCC model of hepatocarcinogenesis the expression level of CYP2E1 gradually decreased with the aggravation of liver lesion [13]. As the downregulation of CYP2E1 occurred earlier than the onset of tumor occurrence, it was possible that decreased CYP2E1 might play a role in hepatocarcinogenesis. Indeed, decreased CYP2E1 was associated with poorly differentiated HCC and poor prognosis of patients with HCC [14]. These findings imply that high expression of CYP2E1 might have an inhibitory role in HCC development. In fact, elevated CYP2E1 level by alcohol induction [15], ectopic expression [16, 17], or exposure to TSA [18] is cytotoxic to HCC cells and inhibits their rapid proliferation. Thus, CYP2E1 may play multiple roles at different stages during hepatocarcinogenesis, which merits more detailed investigation.

CYP2E1 has been shown to be involved in many signaling pathways, such as the AKT [19], PPARα[20], EGFR/c-Raf, ERK1/2 [21], MAPK [22], Nrf2/HO-1, NF-κB [23], and the JNK pathways [24]. Wnt/β-catenin signaling is frequently deregulated in HCC, where activation of Wnt leads to HCC initiation, progression, invasion, and metastasis [25]. Up to 70% of HCCs exhibit increased Wnt/β-catenin signaling, and *CTNNB1* (β-catenin) mutations are elevated with HCC progression [26]. However, the relationship between CYP2E1 and Wnt/β-catenin signaling is unknown.

The current study was designed to unravel the roles of CYP2E1 in the pathogenesis of HCC. We investigated: (1) whether CYP2E1 has a protective role in the malignant biological behavior of HCC; (2) if so, whether CYP2E1 acts as a tumor suppressor by regulating the Wnt/β-catenin pathway in HCC, and (3) the potential links between CYP2E1 suppression and the Wnt/β-catenin pathway in HCC.

## Materials and methods

### Patients and tissue samples

A total of 88 HCC and adjacent non-tumor tissues were collected from patients who underwent liver cancer hepatectomy at the Affiliated Cancer Hospital of Zhengzhou University (Zhengzhou, China) from July 2016 to December 2017, of which 81 samples were paired. Detailed information of the sample refers to Additional file 1: Table S1. The ethics committee of Zhengzhou University approved the protocol (18 March 2016; Approved No: 2016-0177) and all the patients gave written informed consent. In addition, all specimens were subjected to RT-qPCR after RNA extraction, and 36 and 20 samples were used for western blot and immunohistochemical verification, respectively.

### Cell culture and reagents

Human liver cell lines SMMC-7721, HepG2, MHCC-97H, Huh7, Bel-7402 were donated by Dr. Tingting Liu (Sino-American Hormelian Cancer Research, Zhengzhou, P.R. China) and Prof. Ping Xu (State Key Laboratory of Proteomics, Beijing Proteome Research Center, National Center for Protein Sciences, Beijing, P.R. China). HCC-LM3 was purchased from China Center for Type Culture Collection (Wuhan, China). All cell lines were cultured at 37 °C with 5% CO_2_ and grown in Dulbecco's modified Eagle's medium (BI, Shanghai) supplemented with 10% fetal bovine serum, 100 U/ml penicillin and 100 μg/ml streptomycin. 3-Methyladenine (3-MA) was provided by Santa Cruz Biotechnology (sc-205596). *N*-Acetyl-l-cysteine (NAC) and the CYP2E1 inhibitor clomethiazole (CMZ) was purchased from Yuanye Bio-Technology (Shanghai, China).

### RT-qPCR

RT-qPCR was performed according to our previous study [6] and the primers used are listed in Additional file 1: Table S2. Briefly, total RNA was extracted by the RNAiso Plus kit (Takara Bio Inc.). The PrimeScript RT reagent kit (Takara Bio Inc) was used to synthesize the cDNA from 1 μg of total RNA. The transcript levels of the target genes were measured by an ABI 7500 Fast Real-Time PCR system. *GAPDH* was used as a reference gene.

### Western blot

Western blot assays were performed as described recently [27]. Briefly, cells were lysed on ice and proteins were fractionated by SDS-PAGE. Then the protein was transferred onto a PVDF membrane, which was incubated with the indicated antibody and ECL solution. The signal was visualized with an ECL detection system. Antibodies used include anti-CYP2E1 (ab28146, from Abcam, UK); anti-c-Myc (10828-I-AP), Goat anti-rabbit IgG (HL) (SA00001-2), anti-β-Catenin (66379-1-Ig), goat anti-mouse IgG (HL) (SA00001-1), anti-LEF1 (14972-1-AP) (from Proteintech, Wuhan, China); anti-GAPDH (6C5, sc-47724), anti-β-actin (C4, sc-47778), anti-Dvl2 (D-11, sc-390303), anti-TCF-4 (D-4, sc-166699), anti-KLHL12 (D-1, sc-514874), anti-Ub antibody (P4D1, sc-8017) (from Santa Cruz Biotechnology, Inc, USA); anti-E-cadherin (WL01482), anti-Twist (WL00997), anti-N-cadherin (WL01047), anti-Snail (WL01863), anti-Vimentin (WL01960), and anti-Slug (WL01508) from Wanlei Life Sciences, Shenyang, China.

### Gene expression manipulations

Vector lentivirus and lentivirus encoding human CYP2E1 (EX-I0304-Lv201 and EX-NEG-Lv201) were provided by GeneCopoeia (Guangzhou, China). Control or CYP2E1 vectors were first mixed with packaging plasmids (Lenti-Pac™ HIV, GeneCopoeia company) and then were co-transfected into HEK293T cells by Lipofectamine 2000 (Thermo Fisher Scientific Inc). Culture supernatants were collected and purified (LPR-LCS-01, GeneCopoeia, Guangzhou, China) at 48 h after transfection. In the presence of 8 μg/ml polybrene, MHCC-97H and SMMC-7721 cells were infected with the virus particles and finally, puromycin was used to select the infected cells. The overexpression of CYP2E1 in transfected cells was verified by RT-qPCR and western blot. To overexpress Dvl2, the full-length Dvl2 plasmid (GV417-Dvl2) was transiently infected into the CYP2E1 stable cells by VigoFect (Vigorous Biotechnology).

### Cell migration assay

For cell migration assay, HCC cells were plated in 6-well plates and grown to confluence. The cell monolayer was scratched with a sterile 10 μl pipette tip and exchanged with serum-free DMEM after washing twice with PBS. The images of the wounds were recorded at the same position using an inverted microscope (Nikon ECLIPSE TS100, Japan) at 0 h, 24 h, 48 h and 72 h, respectively. The width between wounds was detected by Image J software (National Institutes of Health).

### Cell invasion assay

For cell invasion capacity, the Transwell chamber was coated with 100 μl 1: 8 diluted Matrigel (BD BioCoat, Corning, USA) and incubated for 5 h. Next, 5 × 10^4^ cells in serum-free medium (200 μl) was seeded at the upper chamber, and 600 μl of DMEM medium containing 10% FBS was added to the lower chamber. After 36 h incubation at 37 °C, the matrigel in the upper chamber was gently removed using a cotton swab. The invaded cells were fixed with paraformaldehyde (4%) and stained with DAPI. The cells were photographed using fluorescence microscopy and counted from 5 random fields of microscope.

### CCK-8 assay

200 μl of cell suspension (5 × 10^3^ cells) were added into 96-well plates and incubated at 37 °C. At the appointed time, each well was added with 10 μl CCK-8 reagent (Dojindo Laboratories, Japan) and the absorbance at 450 nm was determined by a microplate spectrophotometer (Bio Tek, USA).

### TOPFlash reporter assay

HCC-Ctrl or HCC-CYP2E1 cells were seeded in 24-well plates and incubated for 24 h at 37 °C. Then the cells were transfected with TOPFlash plasmid (800 ng/well) and pRL-TK plasmid (50 ng/well) using VigoFect reagent (Vigorous Biotechnology). The Dual Luciferase Reporter Assay Kit (Promega, Madison, WI, USA) was employed to to measure Luciferase and Renilla activities 48 h after transfection. The luciferase activity of each sample was normalized with the respective Renilla activity.

### Detection of reactive oxygen species (ROS)

Cells grown to the appropriate 70–80% confluence in 6-well plates were stained with 20 μmol/l dihydroethidium (DHE, ABP Biosciences, Germany) for 1 h in the dark to assess the ROS level. ROS generation was indicated by red fluorescence and detected at Em: 485 nm and Ex: 610 nm with a fluorescence microscope.

### Co-immunoprecipitation (co-IP) experiment

Protein extraction and quantification are as described for western blotting. Briefly, total protein (800 μg) was precleared with Protein A/G agarose (sc2003; Santa Cruz) at 4 ℃ for 30 min to remove non-specific protein. After centrifugation, the precleared supernatant was incubated with 2 μg of primary antibodies at 4 ℃ overnight and then 40 μl Protein A/G agarose was added and incubated at 4 ℃ for 4 h. Next, the agarose beads were collected by centrifugation at 3000 rpm and extensively washed with RIPA lysis buffer. Finally, the bound proteins were mixed with loading buffer and subjected to western blotting analysis. Mouse IgG was used as a negative control.

### Immunohistochemistry

Liver tissues were embedded with paraffin, sectioned, dewaxed, and dehydrated with gradient ethanol. After antigen repair, the slides were incubated with CYP2E1 antibody (ab28146, Abcam; 1:125 dilution) at 4 °C overnight. Then, the slides were washed with TBST and incubated with HRP-conjugated secondary antibody polymer (SA00001-2, Proteintech, Wuhan, China; 1:250 dilution) followed by the addition of DAB solution and counterstained with hematoxylin.

### Immunofluorescence cell staining

The cells seeded on the coverslips in the 24-well plates were cultured for 24 h. After fixing with 4% paraformaldehyde and permeabilizing with 0.5% Triton X-100, the cells were incubated with mouse anti-E-cadherin (2:250 dilution, AF0138, Beyotime) and rabbit anti-vimentin (2:250 dilution, 10366-1-AP, proteintech) at 4 ℃ overnight and then with fluorescence secondary antibodies (CoraLite 594, SA00013-3, goat anti-mouse IgG, Proteintech, 1:500 dilution; CoraLite 594, SA00013-4, goat anti-rabbit IgG, Proteintech, 1:500 dilution) for 30 min at 37 ℃. DAPI was used to stain the nuclei for 5 min and coverslips were mounted. Then, the cells were observed by the fluorescence microscope (OLYMpusBX43).

### Tumor xenografts model

Four-week-old male BALB/c nude mice were provided by the Beijing Vital River Laboratory Animal Technology Company. After habituation for 3 days, 100 μl of MHCC-97H-GFP or MHCC-97H-CYP2E1 cell suspension (1 × 10^7^ cells/ml) were injected subcutaneously into the right flanks of mice. Tumor volumes was recorded every 3 days. Tumor volume was calculated with the formula of [0.5 × length × width^2^]. At 35 days after injection, the nude mice were euthanized and tumors were collected for the subsequent orthotopic liver tumor model. These animal experiments were approved by the Animal Ethics Committee of Zhengzhou University and carried out in accordance with the guidelines of animal ethics.

### Mouse liver orthotopic transplantation model

The tumors taken from the above xenograft nude mice were immediately minced into small pieces (about 1.5 mm × 1.5 mm × 1.5 mm). The left lobe of the liver of the 4.5-week-old male mice was extruded under anesthetization and a tunnel was made by a 10 ml syringe needle. Then 2 pieces of the tumors were deposited into the left lobe through the tunnel. Subsequently, the left lobe of the liver was returned to the abdominal cavity, and the muscle and skin were sutured. Ten weeks after the operation, the livers were removed and orthotopic tumors were counted.

### Pulmonary metastasis model

200 μl of MHCC-97H-GFP or MHCC-97H-CYP2E1 cell suspension (1 × 10^6^ cells/ml) were injected into the tail vein of 4.5-week-old mice. At the 9th week the nude mice were euthanized and the lungs removed. The lungs were fixed with 4% paraformaldehyde, embedded in paraffin, and cut into slices. The slices were moved onto glass slides, dewaxed, rehydrated in ethanol solution and followed by the addition of hematoxylin and eosin to determine the metastasis of cancer cells.

### Statistical analyses

SPSS version 21.0 and GraphPad Prism 5.0 were used to perform statistical analysis and generate graphs, respectively. The Kolmogorov–Smirnov and Shapiro–Wilk methods were used to assess the normality of the data. For normal distribution data, a two-tailed Student’s *t*-test was used for pairwise comparisons. The Mann–Whitney *U* test was used to compare two groups with non-normal distribution data. The overall survival data were analyzed by Kaplan–Meier plots and the cut-off value for defining the subgroups was the median expression level of CYP2E1 mRNA. The sample was then divided into two groups. One group consisted of samples with CYP2E1 expression levels higher than the median, and the other group consisted of the remaining samples. A *P* value (two-tailed) < 0.05 indicated statistically significant.

## Results

### CYP2E1 is downregulated in HCC tissues and the low expression of CYP2E1 is negatively correlated to the malignant clinicopathological features

**Fig. 1:**
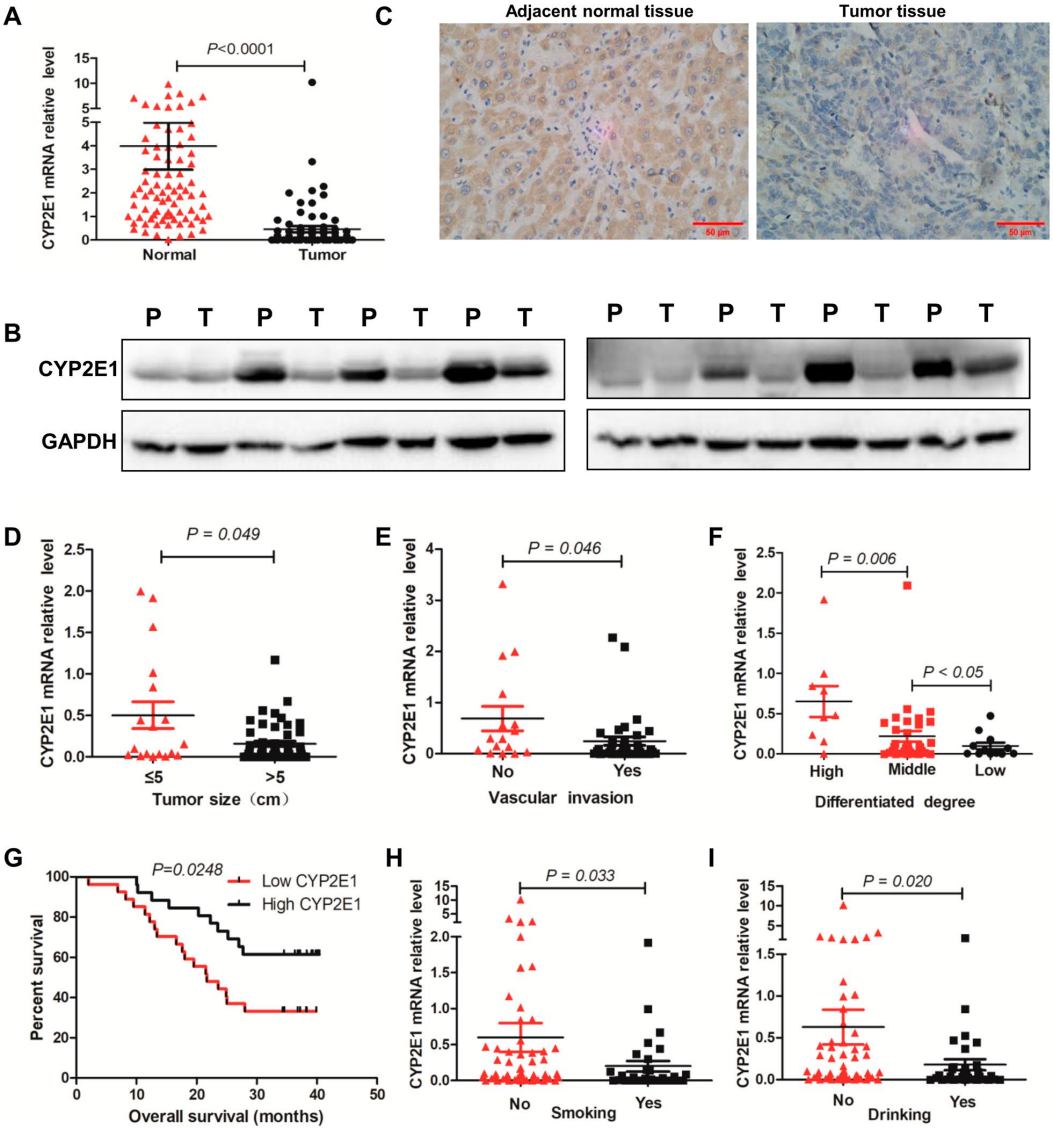
CYP2E1 level is downregulated in HCC and the low expression of CYP2E1 is negatively related to malignant clinicopathologic features of HCC patients. **A** CYP2E1 mRNA levels in adjacent nontumor tissues and HCC tissues were analyzed by RT-qPCR (n = 88). **B** CYP2E1 protein levels in paired adjacent nontumor (N) and tumor (T) tissues measured by western blot assay. **C** Representative images of IHC staining for CYP2E1 in paired adjacent nontumor and tumor tissues. Bar = 50 μm. **D**–**H** The effect of tumor size (**D**), vascular invasion (**E**), degree of differentiation (**F**), smoking (**H**), and alcohol consumption (**I**) on CYP2E1 mRNA level in tumor tissues. **G** Kaplan–Meier curves for overall survival in HCC patients with variable CYP2E1 expression

The expression level of CYP2E1 mRNA in 88 human HCC specimens was determined by qPCR. The results show that CYP2E1 expression in the tumor tissues was markedly down-regulated compared with the adjacent nontumor tissues (Fig. 1A). Downregulation of CYP2E1 protein was also confirmed by western blotting and IHC assays in 77.8% (28/36) and 75% (15/20) of the tumor tissues, respectively (Fig. 1B, C). Patients with larger tumor sizes (> 5 cm) or vascular invasion showed substantially reduced CYP2E1 expression compared with those with smaller tumor sizes (≤ 5 cm) (*P* = 0.049) (Fig. 1D) or without vascular invasion (*P* = 0.046) (Fig. 1E). More strikingly, the expression level of CYP2E1 gradually decreased from well and moderately differentiated to poorly differentiated tumor tissues (*P* < 0.05) (Fig. 1F). Kaplan–Meier analysis revealed that HCC patients with low expression of CYP2E1 demonstrated a lower overall survival compared with those with high expression of CYP2E1 (median survival 21.7 months versus > 40.4 months) (P = 0.0248) (Fig. 1G). These data indicate that low CYP2E1 expression may play an important role in promoting HCC malignant progression and CYP2E1 can be used as a potentially valuable biomarker for predicting the prognosis of HCC. In addition, we found that smoking and drinking decreased the expression level of CYP2E1 in tumor tissues, respectively (Fig. 1H, I).

### Overexpression of CYP2E1 inhibits the proliferation, migration and invasion of HCC cell both in vitro and in vivo

**Fig. 2:**
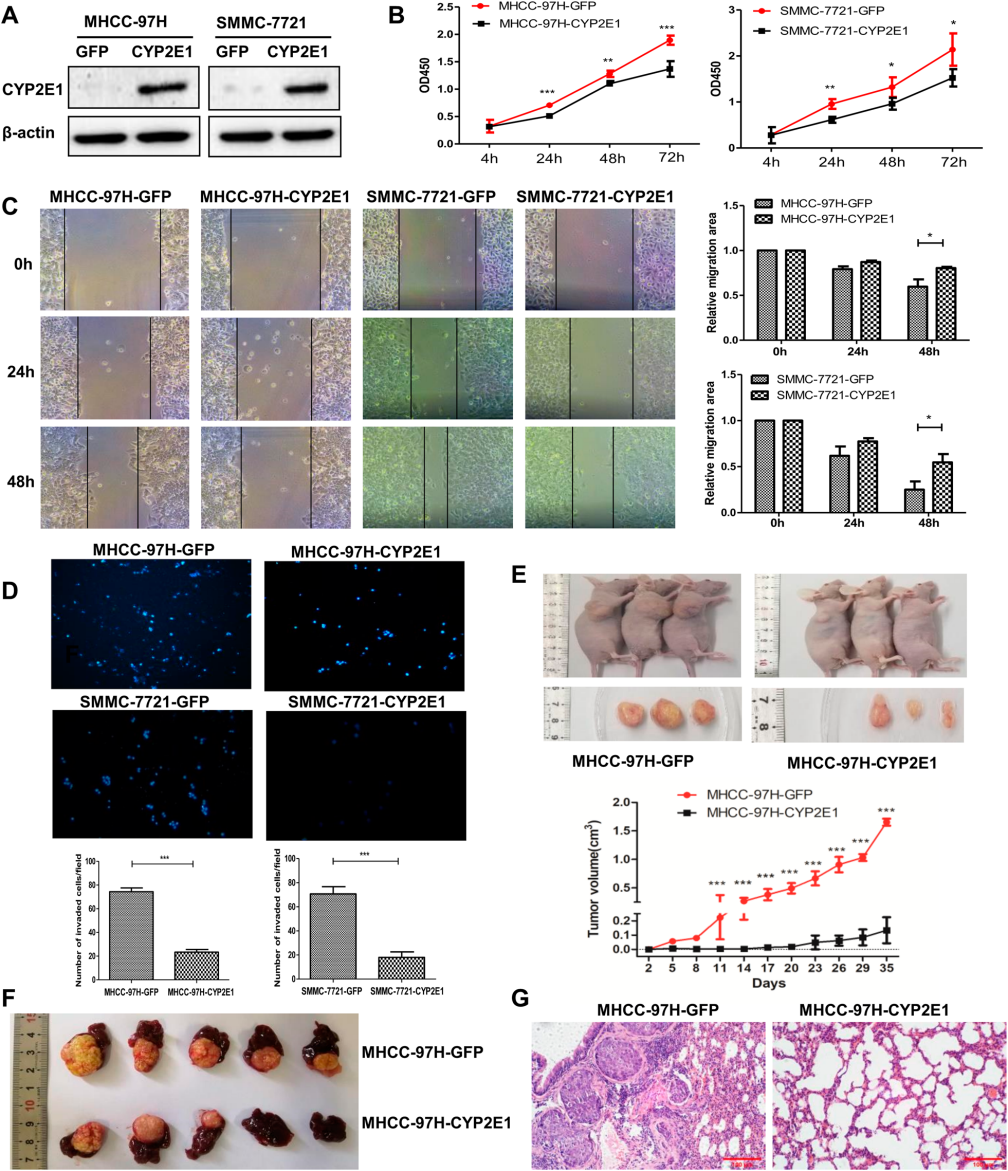
Overexpression of CYP2E1 inhibits the proliferation, migration, and invasion of HCC cells in vitro and in vivo. **A** CYP2E1 protein expression was evaluated by western blotting in control and CYP2E1-overexpressing HCC cells. **B** The effect of CYP2E1 overexpression on cellular survival as determined by CCK-8 assays. **C** Representative images from scratch wound assays of MHCC-97H-CYP2E1 (left panel), SMMC-7721-CYP2E1 (middle panel), and the respective control cells (×100). Statistical graphs of scratch wound assays (right panel). **D** Representative images and statistical analysis (down panel) of transwell assays for MHCC-97H-CYP2E1, SMMC-7721-CYP2E1, and the respective control cells (×100). **E** Image and growth curve of xenograft tumors in nude mice injected with the indicated cells (n = 3 in each assay). **F** Images of the liver orthotopic transplantation tumors in nude mice implanted with the subcutaneous tumors from MHCC-97H-CYP2E1 or MHCC-97H-GFP cells. **G** Representative H & E images of lung tissue slides of nude mice injected by tail vein with MHCC-97H-CYP2E1 and MHCC-97H-GFP. The data in **B**–**E** are presented as the means ± sd. *P ≤ 0.05; **P ≤ 0.01; ***P ≤ 0.001 vs. control group

The expression of CYP2E1 mRNA was low in all six HCC cell lines we examined (Additional file 2: Fig. S1A). We then constructed stable expression of CYP2E1 in MHCC-97H and SMMC-7721 cells, which showed the lowest expression of endogenous CYP2E1, using a lentiviral system (MHCC-97H-CYP2E1, SMMC-7721-CYP2E1) (Fig. 2A and Additional file 2: Fig. S1B). Cell Counting Kit-8 assays, wound healing and Transwell assays with MHCC-97H-CYP2E1, SMMC-7721-CYP2E1 cells revealed that the exogenous expression of CYP2E1 significantly inhibited cell proliferation, migration and invasion in comparison with that of control cells (MHCC-97H-GFP, SMMC-7721-GFP) (Fig. 2B–D).

To confirm the consequences of CYP2E1 overexpression in vivo, we first injected MHCC-97H-CYP2E1 and MHCC-97H-GFP cells into the right dorsal flank of nude mice. Tumors were visible at day 5 with MHCC-97H-GFP cells and grew rapidly after the 8th day of injection, whereas the group injected with MHCC-97H-CYP2E1 cells developed visible tumors on the 17th day of injection and the tumors grew very slowly. At day 35 after injection, two groups of nude mice were euthanized and the tumors that developed from MHCC-97H-CYP2E1 cells were significantly smaller and lighter than tumors from the control group (Fig. 2E). Next, the subcutaneous tumors from the above two groups of nude mice were used for the liver orthotopic transplantation tumor model experiment. After 10 weeks of implantation, two groups of nude mice were euthanized and the livers removed. The results show that tumors formed in the livers of all 5 mice in the control group, whereas tumors were only observed in 3/5 nude mice with MHCC-97H-CYP2E1 subcutaneous tumor implantation and were significantly smaller (Fig. 2F). Further, lung metastasis was also detected using MHCC-97H-CYP2E1 and MHCC-97H-GFP by tail vein injection. H&E staining of lung tissue sections revealed that fewer and smaller micrometastatic lesions in lungs from nude mice injected with CYP2E1-overexpressing cells compared with those injected with control cells (Fig. 2G). Therefore, the above results demonstrate that CYP2E1 is capable of suppressing the aggressive and highly metastatic phenotype of HCC both in vitro and in vivo.

### Overexpression of CYP2E1 inhibits the Wnt/β-catenin signaling pathway by promoting the ubiquitin-mediated degradation of Dvl2

**Fig. 3:**
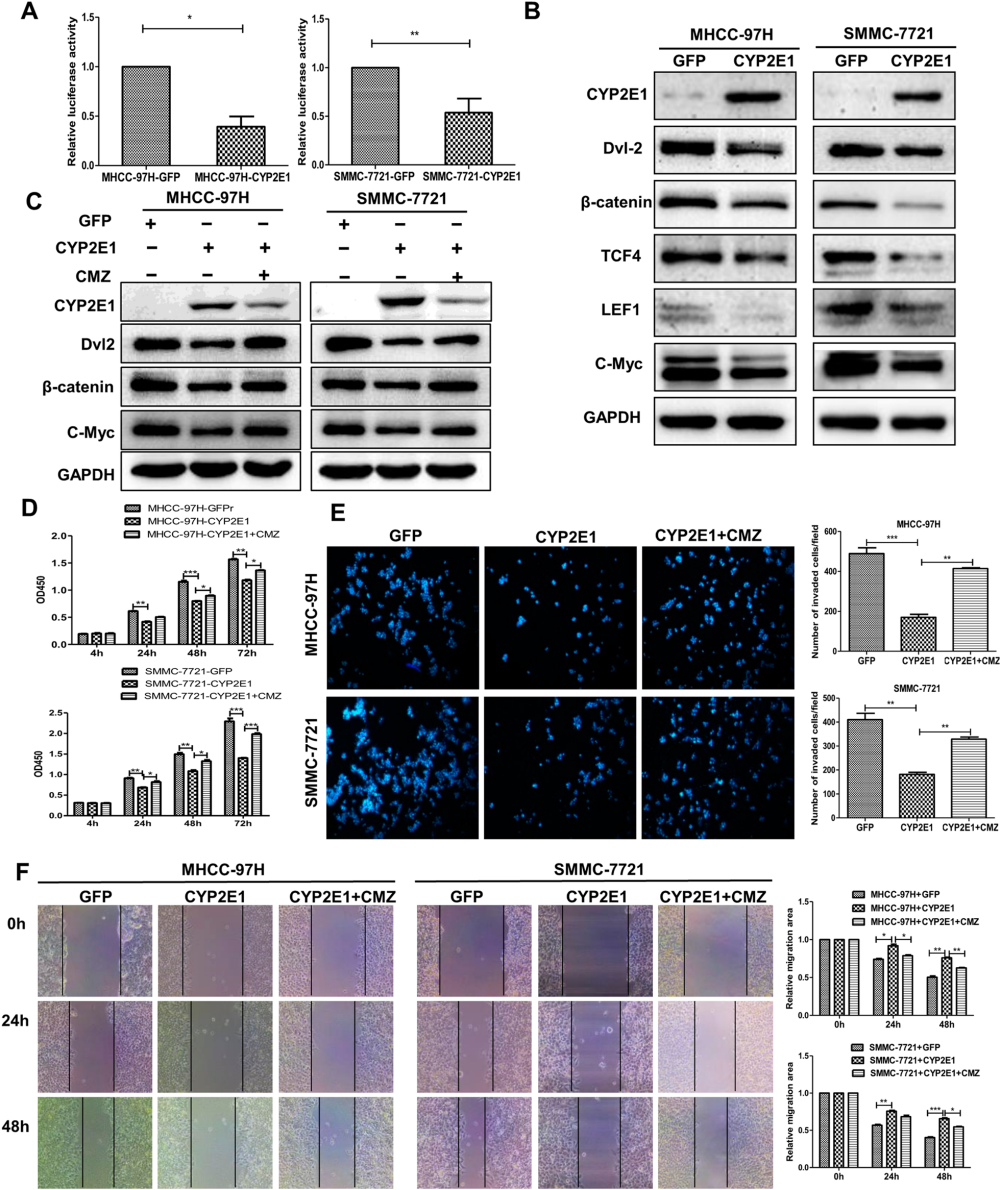
CYP2E1 inhibits Wnt/β-catenin signaling via reducing the Dvl2 protein level. **A** Luciferase activity of TOPFlash in MHCC-97H-CYP2E1, SMMC-7721-CYP2E1 and their respective control cells. Data are shown as mean ± sd (n = 3 in each assay). *P ≤ 0.05, **P ≤ 0.01 vs. control cells. **B** Protein level of Dvl2, β-catenin, TCF4, LEF1 and c-Myc (Wnt/β-catenin signaling-related molecules) in control and CYP2E1-overexpressing HCC cells measured by western blot. **C** The protein expression of CYP2E1, Dvl2, β-catenin, and c-Myc in control and CYP2E1-overexpressing HCC cells at 6 h after treatment with the CYP2E1 inhibitor CMZ (40 μM). **D** The OD450 value of MHCC-97H-CYP2E1, SMMC-7721-CYP2E1 and the respective control cells after treatment with the CYP2E1 inhibitor CMZ (40 μM). **E** Representative images and statistical graphs (right panel) of transwell assays for the indicated cells after treatment with the CYP2E1 inhibitor CMZ (40 μM) (×100). **F** Representative images and statistical graphs (right panel) of scratch wound assays of MHCC-97H-CYP2E1, SMMC-7721-CYP2E1 and the respective control cells after treatment with the CYP2E1 inhibitor CMZ (40 μM) (×100). Data in 3D, 3E and 3F are shown as mean ± sd (n = 3–6 in each assay). *P ≤ 0.05, ^**^P ≤ 0.01, ***P ≤ 0.001 vs. CYP2E1

To uncover further the molecular mechanism by which CYP2E1 inhibits the malignant phenotype of HCC, we first performed a TOPFlash reporter assay to observe the effect of CYP2E1 on Wnt/β-catenin signaling. The result demonstrated that CYP2E1 overexpression significantly reduced the β-catenin/T-cell factor 4 (TCF4)-dependent transcriptional activity both in MHCC-97H and SMMC-7721 cells (Fig. 3A). Next, we examined the effect of CYP2E1 on the expression of the important Wnt/β-catenin pathway members in HCC cells and found that the protein levels of Dvl2 (Dishevelled 2), β-catenin, TCF4, LEF1(lymphoid enhancer factor 1) and c-Myc were decreased in SMMC-7721-CYP2E1 and MHCC-97H-CYP2E1 cells compared with their control cells (Fig. 3B). Chlormethiazole (CMZ), a specific inhibitor of CYP2E1, performs its inhibitory action by reducing the protein level of CYP2E1 as well as acting as a noncompetitive inhibitor of CYP2E1 [17, 28]. Treatment with the CYP2E1 inhibitor CMZ (40 μM), which obviously reduced CYP2E1 protein levels, significantly restored the protein levels of Wnt pathway proteins in CYP2E1-overexpression cells (Fig. 3C). Moreover, the inhibitory effect of CYP2E1 overexpression on cell proliferation, invasion and migration was significantly rescued by CMZ (Fig. 3D–F). Thus, we conclude that CYP2E1 negatively regulates the Wnt/β-catenin signaling pathway.

**Fig. 4:**
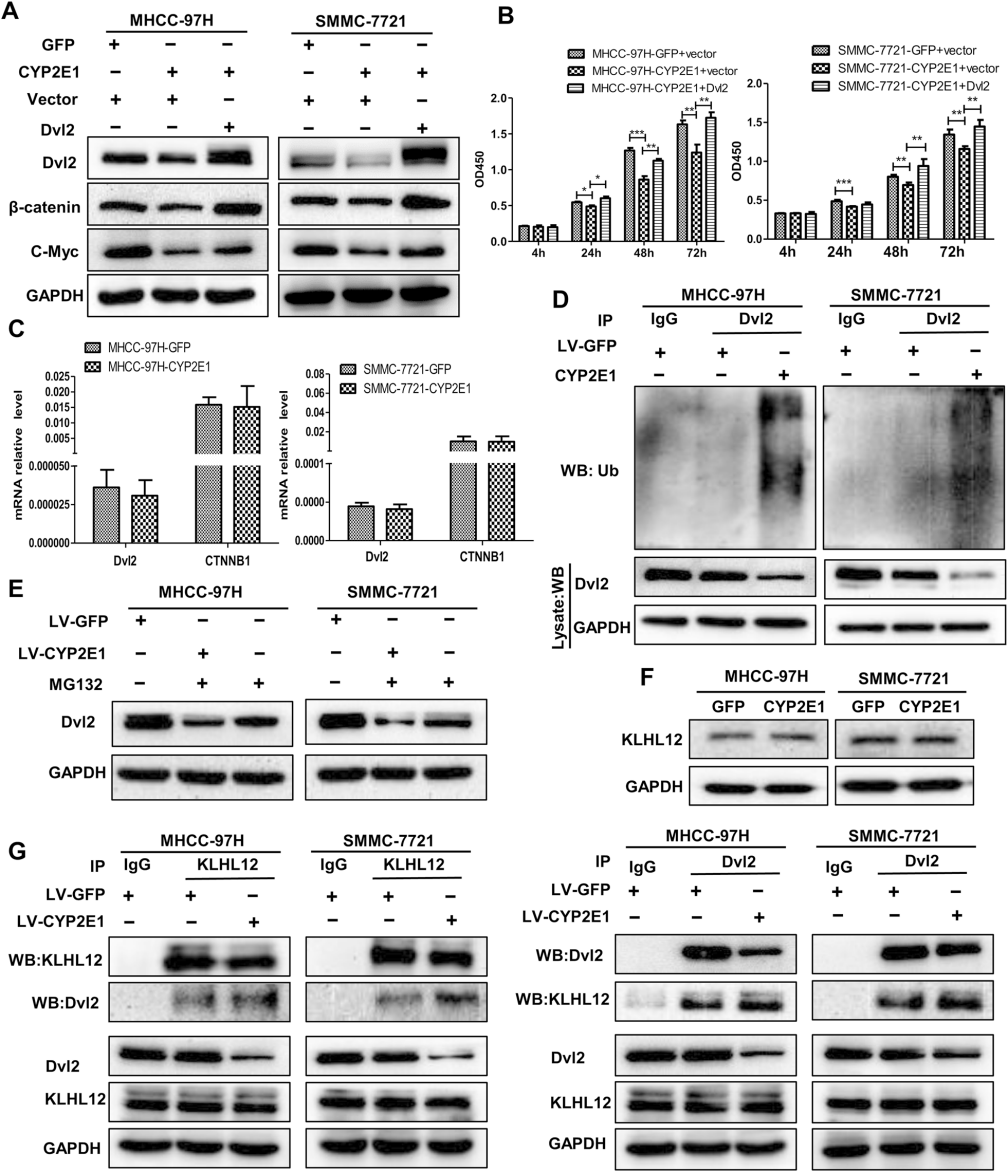
Degradation of Dvl2 in CYP2E1-overexpressing cells is ubiquitin–proteasome pathway dependent. **A** Control or CYP2E1-overexpressing HCC cells were transfected with empty vector or the Dvl2 expression vector. The protein level of Dvl2, β-catenin, and c-Myc were detected by western blot 48 h after transfection. **B** The OD450 value of MHCC-97H-CYP2E1, SMMC-7721-CYP2E1 and the respective control cells after transient transfections with empty vector or the Dvl2 expression vector. ^*^P ≤ 0.05, ^**^P ≤ 0.01, ***P ≤ 0.001 vs. CYP2E1 + vector. **C** The mRNA levels of Dvl2 and CTNNB1 in control and CYP2E1-overexpressing HCC cells were detected by qRT-PCR. **D** Co-immunoprecipitation analysis was applied to determine the ubiquitination level of Dvl2 in control and CYP2E1-overexpressing HCC cells. **E** Western blot analysis of Dvl2 protein levels in control and CYP2E1-overexpressing HCC cells at 1 h after treatment with the proteasome inhibitor MG132 (10 mM). **F** The protein level of KLHL12 in MHCC-97H-CYP2E1, SMMC-7721-CYP2E1 and the respective control cells was evaluated by western blot. **G** The interaction between Dvl2 and KLHL12 was determined by reciprocal co-immunoprecipitation analysis in control and CYP2E1-overexpressing HCC cells

Dvl2 plays a core role in the Wnt signaling pathway [29]. The above results suggest that CYP2E1 may exert its Wnt-regulatory function via Dvl2. We ectopically expressed Dvl2 and found that forced expression of Dvl2 restored the protein level of Dvl2, β-catenin, and c-Myc (Fig. 4A) as well as the inhibitory effect of CYP2E1 overexpression on cell proliferation in CYP2E1-overexpressing cells (Fig. 4B). Collectively, these results indicate that CYP2E1 inhibits the malignant phenotype of HCC by negatively regulating Wnt signaling via downregulation of Dv12. Interestingly, overexpression of CYP2E1 in HCC cells did not affect the mRNA level of Dvl2 and β-catenin (Fig. 4C), which suggests that the downregulation of Dv12 by CYP2E1 may result from protein stabilization. Previous studies revealed that the ubiquitin–proteasome pathway regulated Dvl2 degradation [30]. We then speculated that CYP2E1 may function in HCC cells similarly. Indeed, overexpression of CYP2E1 markedly increased the protein polyubiquitination of Dvl2 (Fig. 4D). We then treated HCC cells with the proteasome inhibitor MG132 (10 mM) for 1 h and found that the Dvl2 protein level in the CYP2E1-overexpressing cells was significantly restored (Fig. 4E), suggesting that the Dvl2 decrease in CYP2E1-overexpressing HCC cells may involve a ubiquitin-dependent pathway. The activation of the ubiquitin–proteasome pathway requires E3 ubiquitin ligase to specifically recognize the substrate molecule. KLHL12 was the identified E3 ubiquitin ligase of Dvl2 [30]. Next, we determined if the degradation of Dvl2 by CYP2E1 was related to KLHL12. Western blot results showed that there was no difference in KLHL12 protein expression levels between the CYP2E1-overexpressing cell and the control cells (Fig. 4F), indicating that CYP2E1 did not affect the protein level of KLHL12. We then performed reciprocal Co-IP assays and the results displayed that Dvl2 interacted with KLHL12 and CYP2E1 overexpression enhanced the interaction between Dvl2 and KLHL12 compared with the control group (Fig. 4G). Taken together, these results indicate that CYP2E1 promotes the ubiquitin-mediated degradation of Dvl2 by enhancing the interaction between KLHL12 and Dvl2.

### CYP2E1-mediated inhibition of the HCC phenotype as well as the Wnt/β-catenin signaling pathway is ROS-dependent

**Fig. 5:**
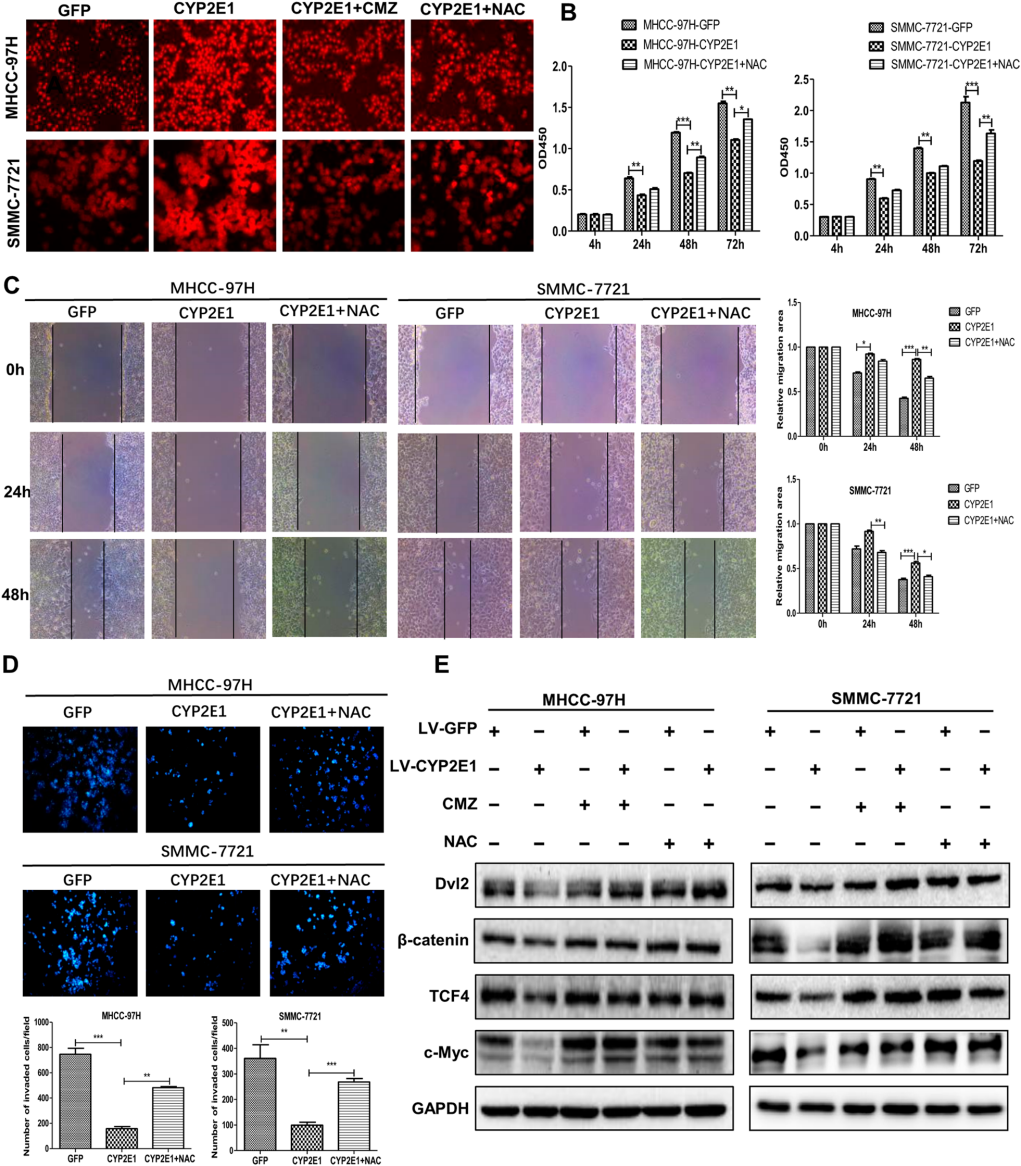
CYP2E1-inhibited Wnt/β-catenin signaling by ROS. **A** ROS level in control and CYP2E1-overexpressing HCC cells detected by fluorescence probe and the change in ROS level after treatment with the CYP2E1 inhibitor CMZ (40 μM) or the antioxidant NAC (10 mM) for 6 h in the indicated cells. **B** The OD450 value of MHCC-97H-CYP2E1, SMMC-7721-CYP2E1 and the respective control cells after treatment with NAC (10 mM) at the indicated time points. **C** Representative images and statistical analysis (right panel) of scratch wound assays of MHCC-97H-CYP2E1, SMMC-7721-CYP2E1 and the respective control cells after treatment with NAC (10 mM) (×100). **D** Representative images and statistical analysis (down panel) of transwell assays of MHCC-97H-CYP2E1, SMMC-7721-CYP2E1, and the respective control cells after treatment with NAC (10 mM) (×100). **E** After treatment with the CYP2E1 inhibitor CMZ (40 μM) or the antioxidant NAC (10 mM) for 6 h, the protein levels of Dv1-2, β-catenin, TCF4 and c-Myc in MHCC-97H-CYP2E1, SMMC-7721-CYP2E1 and the respective control cells were evaluated by western blot. Data in 5B, 5C and 5D are shown as mean ± sd (n = 3–6 in each assay). *P ≤ 0.05, ^**^P ≤ 0.01, ***P ≤ 0.001 vs. CYP2E1

A unique property of CYP2E1 is to produce reactive oxygen species (ROS). We investigated whether CYP2E1-mediated inhibition of the HCC phenotype as well as Wnt/β-catenin pathway is ROS-dependent. An increased level of intracellular ROS was seen in both MHCC-97H-CYP2E1 and SMMC-7721-CYP2E1 cells in comparison with their corresponding control cells (Fig. 5A). Treatment of the control group and CYP2E1-expressing cells with the CYP2E1 inhibitor CMZ (40 μM) or the antioxidant NAC (10 mM) for 6 h showed that the ROS content of CYP2E1-expressing cells decreased significantly after CMZ or NAC treatment (Fig. 5A and Additional file 3: Fig. S2). At the same time, the inhibitory effect of CYP2E1 on cell proliferation, migration and invasion was significantly rescued by NAC (Fig. 5B–D). In addition, the protein levels of Dvl2, β-catenin, TCF4 and c-Myc in CYP2E1-expressing cells was significantly reversed by treatment with CMZ or NAC (Fig. 5E). These results indicate that CYP2E1-mediated inhibition of the HCC phenotype as well as the Wnt/β-catenin signaling pathway is ROS-dependent.

### Overexpression of CYP2E1 inhibits EMT in HCC cells

**Fig. 6:**
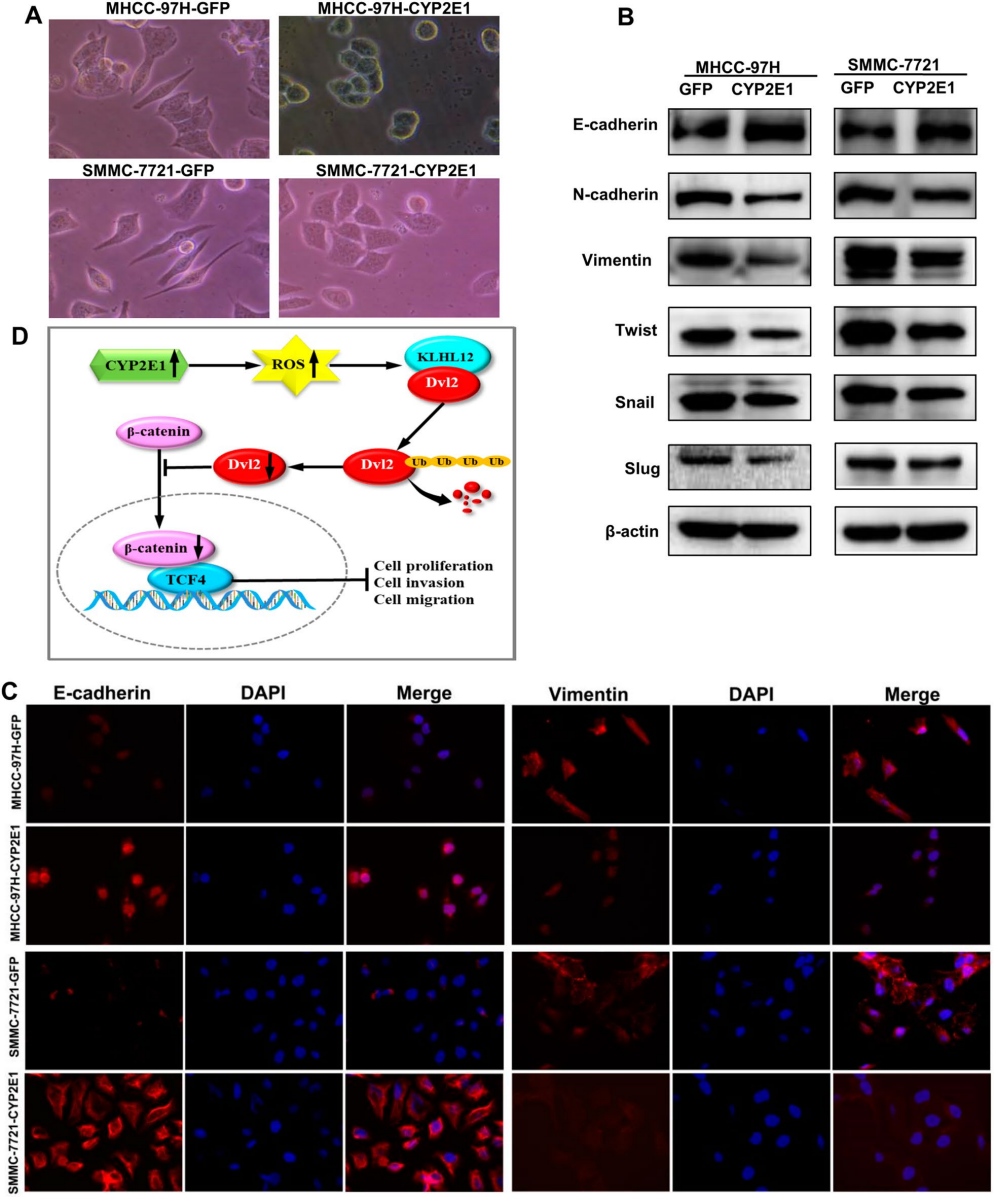
Overexpression of CYP2E1 inhibits EMT in HCC cells. **A** Representative images of the morphological changes associated with EMT of MHCC-97H-CYP2E1, SMMC-7721-CYP2E1 and the respective control cells (×100). **B** Protein levels of EMT markers (E-cadherin, vimentin and N-cadherin) as well as EMT-related transcription factors (Twist, Slug and Snail) of the indicated cells detected by western blot. **C** Typical images of immunofluorescent staining for E-cadherin and vimentin in MHCC-97H and SMMC-7721 cells (×100). **D** Proposed model of the tumor suppressor role of CYP2E1 in HCC. Overexpression of CYP2E1 induces ROS accumulation and promotes the ubiquitin-mediated degradation of Dvl2, thereby, inhibiting the Wnt/β-catenin signaling and playing a suppressive role in HCC

Interestingly, CYP2E1-overexpressing cells show an epithelial cobblestone-like morphology (Fig. 6A). Considering the changes in the morphology of these cells and the function of CYP2E1, we speculate that CYP2E1 may inhibit the EMT of cells. Indeed, overexpression of CYP2E1 increased the expression of epithelial marker (E-cadherin) and decreased the expression of mesenchymal marker (N-cadherin and vimentin) in MHCC-97H and SMMC-7721 cells (Fig. 6B). The immunofluorescence results also exhibited that the expression of E-cadherin increased, while the expression of vimentin decreased in CYP2E1-overexpressing HCC cells (Fig. 6C). In addition, the protein level of EMT-associated transcriptional factors, including Twist, Snail and Slug, were significantly decreased in CYP2E1-expressing cells (Fig. 6B). Thus, overexpression of CYP2E1 inhibits EMT in HCC cells.

## Discussion

Our knowledge of the role of CYP2E1 in hepatocarcinogenesis is based on epidemiological and animal studies, with a primary focus on the role of CYP2E1 in the metabolic activation of procarcinogens; few studies have directly assessed the effects of CYP2E1 on HCC malignant phenotypes. Ho et al. [14] reported that of CYP2E1 expression was significantly downregulated in HCC tissues and might serve as an independent prognostic factor for disease-free survival. Similar findings were reported in some earlier studies [11, 12]. In addition, several reports have proposed that CYP2E1 overexpression is cytotoxic to the tumor cells and inhibits rapid cell proliferation upon ethanol treatment [31, 32]. Here, our results show that the expression of CYP2E1 is decreased in HCC tissues, and low expression of CYP2E1 is associated with larger tumor diameter, vascular invasion, tumor differentiation, and short survival time in patients with HCC. Ectopic expression of CYP2E1 inhibited the proliferation, invasion and migration of HCC cells both in vivo and in vitro. Therefore, a decreased level of CYP2E1 might place the HCC cells at an advantage and favor HCC progression. These results support the hypothesis that CYP2E1 plays a protective role in the malignant biological behavior of HCC.

Wnt/β-catenin signaling is an evolutionarily highly conserved pathway and plays decisive roles in development and tissue homeostasis. Aberrant activation of the Wnt/β-catenin signaling cascade is one of the most frequent molecular events in HCC [33] and conduces to the initiation, progression, invasion, and metastasis of HCC [25]. When the Wnt ligand binds to its receptor, disheveled (Dvl) is recruited to the ligand-receptor complex, which provides a platform for Axin and GSK3β to bind, resulting in the dissociation of the cytoplasmic destruction complex, preventing β-catenin degradation. β-Catenin then forms a complex with TCF4/LEF1 in the nucleus, leading to the activation of Wnt/β-catenin target genes, such as *c-Myc*, *Axin2*, *LEF1*. Dvl is therefore the central component of Wnt/β-catenin signaling and relays signals from Wnt receptors to β-catenin by rescuing β‐catenin from degradation [10, 29]. Three homologous Dishevelled genes (*Dvl1*, *Dvl2* and *Dvl3*), which exhibit a high degree of similarity, have been found in humans [34]. Increasing evidence indicates that Dvl plays critical roles in the progression of breast cancers, non-small cell lung cancer and astrocytomas [34–36]. A recent study revealed that Dvl2 was overexpressed in HCC tissues and interacted with P62 to promote the invasion and migration of HCC cells [37].

In the present study, CYP2E1 overexpression significantly inhibited Wnt/β-catenin signaling activity in HCC cells and Dvl2 expression level was reduced in CYP2E1-overexpressing HCC cells. Restoration of Dvl2 expression rescued the malignant phenotype of HCC cells. Previous studies have verified that Dvl2 is mainly regulated at the protein level by the ubiquitin–proteasome pathway. We thus speculated that the CYP2E1-blocked Wnt/β-catenin signaling pathway might depend on Dvl2 stability. KLHL12–Cullin-3 ubiquitin ligase was reported to mediate ubiquitination and degradation of Dvl2 under different physiological conditions [30, 38]. Our results demonstrate that overexpression of CYP2E1 promotes the ubiquitination of Dvl2 in HCC cells. Further mechanistic research found that CYP2E1 strengthened the interaction of Dvl2‐KLHL12, thus inducing the ubiquitination and degradation of Dvl2 in CYP2E1-stable cells. Therefore, the present study establishes a link between CYP2E1 and Wnt/β-catenin signaling, and indicates that overexpression of CYP2E1 inhibits Wnt/β-catenin signaling by inducing the destabilization and promoting the degradation of Dvl2.

How does CYP2E1 regulate Wnt/β-Catenin signaling? CYP2E1 is one of the most active ROS-generating CYP isoforms and can produce high levels of ROS [39]. ROS are increasingly being recognized as important signaling molecules involved in the regulation of many signaling pathways [40]. Several studies have revealed the ability of ROS to regulate Wnt/β-catenin signaling [41]. For example, H_2_O_2_ negatively regulates the Wnt pathway by downregulating β-catenin [42]. Combretastatin A-1 phosphate, a microtubule polymerization inhibitor, has outstanding anti-cancer activity against HCC by inducing ROS accumulation and thereby inhibiting the Wnt/β-catenin pathway in HepG2 cells [43].

A recent finding revealed that oxidative stress suppressed distant metastasis by melanoma cells in vivo [44]. Large-scale multicentre clinical trials also showed that antioxidant supplementation not only failed to bring benefit to patients, but was related with a significant increase in cancer incidence [45, 46]. In the current study, ROS level were significantly elevated in CYP2E1-overexpressing HCC cells comparing with the control cells. Treatment with CYP2E1 inhibitor or antioxidant not only restored the expression of proteins associated with the Wnt signaling pathway but also rescued the inhibitory phenotypes in CYP2E1 stable HCC cells. Therefore, we speculate that the inhibitory effect of CYP2E1 on the Wnt/β-catenin pathway is ROS-dependent and downregulation of CYP2E1 is beneficial to HCC cells to cope with the ROS stress and favor cancer growth.

Collectively, our studies provide novel insight into the role of CYP2E1 in HCC, independent of its activation of procarcinogens and mutagens. Ectopic expression of CYP2E1 inhibits cell proliferation, migration, invasion and EMT of HCC cells in vitro and inhibited tumor formation and lung metastasis in nude mice. The protective effect of CYP2E1 is attributed to its ability to manipulate Wnt/Dvl2/β-catenin signaling through ROS accumulation, which provides a potential target for the prevention and treatment of HCC.

## Supplementary Information

**Additional file 1: Table S1. **Donor characteristics of human liver samples. **Table S2.** Primers for quantitative real-time polymerase chain reaction.

**Additional file 2: Fig. S1.** The expression level of CYP2E1 mRNA in HCC cells.

**Additional file 3: Fig. S2.** Statistical graphs of ROS level in control and CYP2E1-overexpressing HCC cells.

## Data Availability

The datasets used and analyzed during the current study are included in the manuscript and the supplementary materials.
