# Supplementary material for: CYP2E1 plays a suppressive role in hepatocellular carcinoma by regulating Wnt/Dvl2/β-catenin signaling
Source: J Transl Med. 2022 May 4;20:194. doi: 10.1186/s12967-022-03396-6 (PMC9066941; doi:10.1186/s12967-022-03396-6)
Supplement: Supplementary file 1 — Additional file 1: Table S1. Donor characteristics of human liver samples. Table S2. Primers for quantitative real-time polymerase chain reaction. [file 12967_2022_3396_MOESM1_ESM.docx]

**Supplementary Tables S1-S2**

**Supplementary Table S1** **Donor characteristics of human liver samples**

| Variables | Group | Number | Percentage (%) |
| --- | --- | --- | --- |
| Gender | Male | 69 | 78.4 |
|  | Female | 19 | 21.6 |
| Age (years) | 20-45 | 23 | 26.1 |
|  | 46-60 | 44 | 50.0 |
|  | 61-75 | 21 | 23.9 |
| Smoking | Non-smoking | 57 | 64.8 |
|  | Smoking | 31 | 35.2 |
| Drinking | Non-drinking | 55 | 62.5 |
|  | Drinking | 33 | 37.5 |
| Medical Diagnosis | HBV-HCC | 74 | 84.1 |
|  | HBV-RHCC | 10 | 11.4 |
|  | HCC | 4 | 4.5 |

HBV-HCC: HBV-related primary hepatocellular carcinoma; HBV-RHCC: HBV-related recurrent hepatocellular carcinoma; HCC: primary hepatocellular carcinoma.

**Supplementary Table S2** **Primers for quantitative real-time polymerase chain reaction**

| Gene | Forward primer (5’→3’) | Reverse (5’→3’) | Fragment Size（bp） |
| --- | --- | --- | --- |
| *GAPDH* | AACAGGGTGGTGGACCTCAT | GGAGGGGAGATTCAGTGTGG | 153 |
| CYP2E1 | TTCAGCGGTTCATCACCCT | GAGGTATCCTCTGAAAATGGTGTC | 77 |
| *Dvl-2* | TCAGCAGCGTCACAGATTCC | GTCTCCCCGCTCATTGCTC | 116 |
| *CTNNB1* | CATCTACACAGTTTGATGCTGCT | GCAGTTTTGTCAGTTCAGGGA | 150 |
