# Supplementary figures and images for: CYP2E1 plays a suppressive role in hepatocellular carcinoma by regulating Wnt/Dvl2/β-catenin signaling
Source: J Transl Med. 2022 May 4;20:194. doi: 10.1186/s12967-022-03396-6 (PMC9066941; doi:10.1186/s12967-022-03396-6)

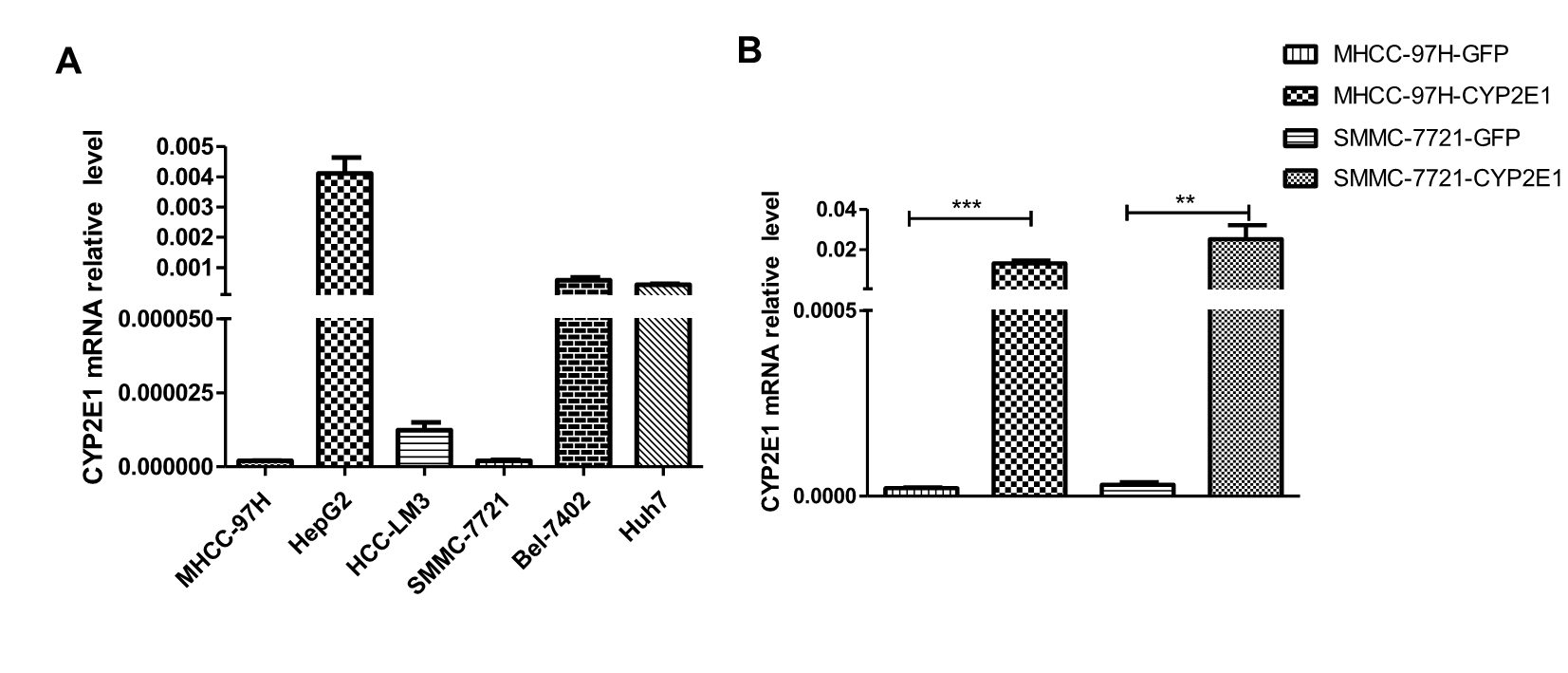

Supplement: Supplementary file 2 — Additional file 2: Fig. S1. The expression level of CYP2E1 mRNA in HCC cells. [file 12967_2022_3396_MOESM2_ESM.tif]

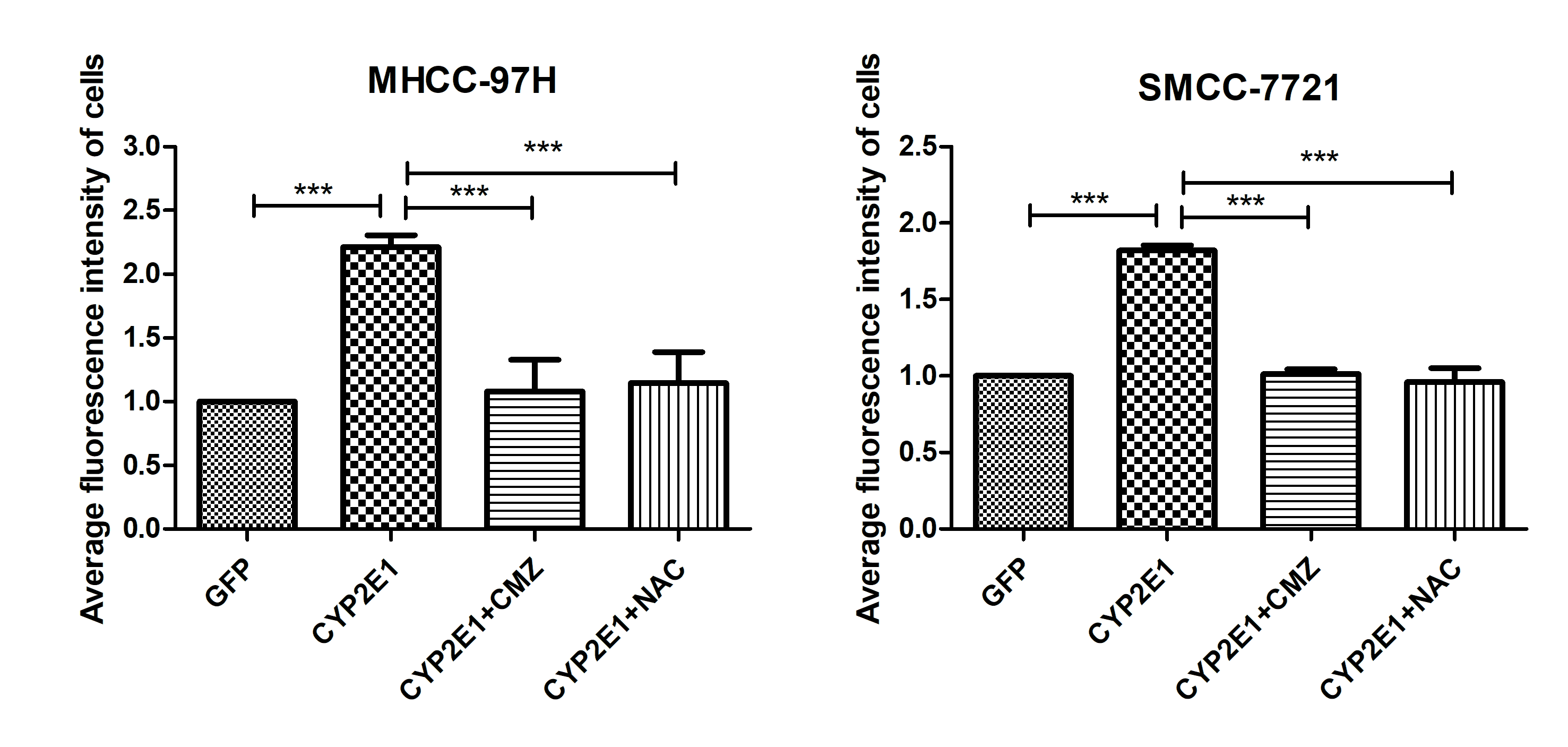

Supplement: Supplementary file 3 — Additional file 3: Fig. S2. Statistical graphs of ROS level in control and CYP2E1-overexpressing HCC cells. [file 12967_2022_3396_MOESM3_ESM.tif]
